# Design and Optimization for a New XYZ Micropositioner with Embedded Displacement Sensor for Biomaterial Sample Probing Application

**DOI:** 10.3390/s22218204

**Published:** 2022-10-26

**Authors:** Minh Phung Dang, Hieu Giang Le, Thu Thi Dang Phan, Ngoc Le Chau, Thanh-Phong Dao

**Affiliations:** 1 Faculty of Mechanical Engineering, Ho Chi Minh City University of Technology and Education, Ho Chi Minh City, Vietnam; 2 Faculty of Mechanical Engineering, Thu Duc College of Technology, Thu Duc City, Ho Chi Minh City, Vietnam; 3 Faculty of Mechanical Engineering, Industrial University of Ho Chi Minh City, Ho Chi Minh City, Vietnam; 4 Division of Computational Mechatronics, Institute for Computational Science, Ton Duc Thang University, Ho Chi Minh City, Vietnam; 5 Faculty of Electrical & Electronics Engineering, Ton Duc Thang University, Ho Chi Minh City, Vietnam

**Keywords:** compliant mechanism, XYZ micropositioner, displacement sensor, optimization, teaching–learning-based optimization

## Abstract

An XYZ compliant micropositioner has been widely mentioned in precision engineering, but the displacements in the X, Y, and Z directions are often not the same. In this study, a design and optimization for a new XYZ micropositioner are developed to obtain three same displacements in three axes. The proposed micropositioner is a planar mechanism whose advantage is a generation of three motions with only two actuators. In the design strategy, the proposed micropositioner is designed by a combination of a symmetrical four-lever displacement amplifier, a symmetrical parallel guiding mechanism, and a symmetrical parallel redirection mechanism. The Z-shaped hinges are used to gain motion in the *Z*-axis displacement. Four flexure right-circular hinges are combined with two rigid joints and two flexure leaf hinges to permit two large X-and-Y displacements. The symmetrical four-lever displacement amplifier is designed to increase the micropositioner’s travel. The displacement sensor is built by embedding the strain gauges on the hinges of the micropositioner, which is developed to measure the travel of the micropositioner. The behaviors and performances of the micropositioner are modeled by using the Taguchi-based response surface methodology. Additionally, the geometrical factors of the XYZ micropositioner are optimized by teaching–learning-based optimization. The optimized design parameters are defined with an *A* of 0.9 mm, a *B* of 0.8 mm, a *C* of 0.57 mm, and a *D* of 0.7 mm. The safety factor gains 1.85, while the displacement achieves 515.7278 µm. The developed micropositioner is a potential option for biomedical sample testing in a nanoindentation system.

## 1. Introduction

Flexure-based/compliant mechanisms have been strongly favored in ultrahigh machinery and precision engineering, e.g., bistable-based switches [1,2], cutting [3], micro grippers [4], harvesters for energy [5], robotics [6,7], and so forth. In precise positioning devices, flexure-based mechanisms have the benefit of a good resolution and being able to produce precisely repeatable motions because the flexure-based mechanisms offer no backlash, no friction, less assembly, and they are cheap.

By taking the aforementioned advantages of compliant mechanisms, many one degree of freedom (DOF) to three-DOF mechanisms have been designed in the field of precision engineering. The one DOF architecture permitted a high-accuracy translation, but it was limited in its practical applications [8]. Then, two DOF architecture was proposed [9,10]. Nevertheless, the one DOF and two DOF architectures have limited applications. From that, three-DOF architecture was developed for more applications [11,12].

Generally speaking, 3-DOF flexure mechanisms have been considered as micro/nano positioners with ultrahigh precision and a compact size. The 3-DOF micro/nano positioning platforms has been extensively used in various applications, such as XYZ micromanipulators [13], XYZ compliant platforms for atomic force microscopy [14], XYθ alignment platforms for wafer photolithography, and so forth. Regarding the actuators, the 3-DOF flexure mechanisms are often exerted by using piezoelectrical actuators (PZT) due to this actuator having an excellent stiffness quality and a high load capacity. However, PZT have a very small stroke. Hence, many types of displacement amplifiers were proposed to amplify the stroke of the PZTs, such as the Scott–Russell mechanism, the lever, and the bridge types [15,16].

In particular, numerous studies have been conducted on designing XYZ compliant micropositioning stages. In the design phase of such mechanisms, a fundamental principle is based on the series, parallel chain, or combined series–parallel types [17]. For example, an XYZ micropositioning stage was designed via using three legs, Leg-X, Leg-Y, and Leg-Z [18]. Each leg was exerted by an actuator. There were three actuators that were used for generating the input displacements and three that were used for responding with the output displacements. In this study, a parallel principle was applied to build a high stiffness for the micropositioning stage. However, one drawback of this stage is the relatively large size in the Z direction. It made a cubic-like micropositioning stage. Based on the parallel-kinematic structure, the XYZ micropositioning platform was built by using three PZTs actuators [19]. The Z directional size was still large; it was about 68.5 mm. The authors employed the bridge-type amplifier to amend the magnitude of the displacement. The output displacements were 94 μm, 120 μm, and 1 μm in the X, Y, and Z directions, respectively. It was found that the X directional displacement differed from the Y and Z displacements. In addition, an XYZ manipulator was designed using three orthogonal limbs in a parallel style, and it used three voice coil actuators for generating three input motions [20]. After the design of the structure, an open-loop dynamic controller was performed. The motions could reach a very large workspace of 2.3 mm × 2.3 mm × 2.3 mm. Nevertheless, the Z-axial size of the manipulator was 148 mm. This size does not have a compact structure yet. Another design of the XYZ micropositioning stage was conducted using the parallel-kinematic design [21]. Three bridge-type amplifiers were employed. To maximize the natural first frequency, the geometrical parameters were optimized by a particle swarm optimization. The optimized frequency was 124.5 Hz. In this study, three PZTs actuators were used, and then, a closed-loop controller was established. This stage could had a workspace of 165.8 μm × 5.4 μm × 6.5 μm. It is observed that there is a difference among the three output displacements in the *X*, *Y*, and *Z*-axes. The *Z*-axial size of this design is relatively large. In this regard, an XYZ parallel platform was proposed, and its geometrical parameters were optimized using the gradient projection method to minimize the parasitic motion [22]. The optimized result achieved a resonant frequency of the first mode shape, which was 56 Hz. It is found that the *Z*-axial size of the platform is also large. Another design of the XYZ mechanism for the manipulator was suggested [23]. The rhombic-type amplifier was applied to amend the magnitude of the travel of the PZTs. The workspace was 112 μm × 112 μm × 123 μm. To produce a large stroke and a high stiffness quality, an XYZ mechanism with a parallel structure was proposed. The XYZ output stroke could reach up to 10 mm × 10 mm × 10 mm. Despite the large workspace, the overall size of the mechanism was not compact [24]. In addition, an XYZ micropositioning platform was designed to permit a large workspace of 10.39 μm × 15.43 μm × 15.55 μm [25]. In this paper, the authors employed three PZTs actuators. This led the complex control of it and a high cost. Recently, a few studies on the XYZ micropositioning stages have been performed. For example, an XYZ flexure mechanism with a parallel structure was actuated by three PZTs which could reach a large space and were costly [26]. The output reach motions of this mechanism achieved up to 141 μm × 141 μm × 141 μm through the parameter optimization by the particle swarm optimization. Regarding the simple control of it, an XYZ manipulator with three same output displacements was developed; the stroke could reach 141 μm × 141 μm × 141 μm, but it employed three PZTs actuators [27]. To produce a large stroke, a lever-combined bridge amplifier was integrated into the XYZ micropositioning stage [28]. The workspace of this mechanism could reach 128.1 μm × 131.3 μm × 17.9 μm. Although numerous studies have focused on designing new structures for the XYZ micropositioning stages, but the existing designs are still complicated in their structure, and the output displacements in the *X*, *Y*, and *Z* axes are not similar. Moreover, the previous studies almost used three PZTs actuators to actuate the input motions, and a parallel-kinematic style was applied in the design stage. These problems caused there to be a large size of the working space, especially in the *Z*-axial size as well as a more complicated control process. For the in situ micro/nanoindentation testing using the scanning electron microscope (SEM), it was difficult for the existing micropositioning stages to be attached using the SEM to probe the properties of the material samples. Because the large size and the usage of three PZTs, the cost of them is related to their relatively high manufacturing costs. In addition, the output stroke in the X, Y, and Z directions are not the same, and the practical control issue faces more difficult challenges.

To realize the accurate travel direction or the force of it, the existing micropositioning stages have utilized displacement laser sensors, capacitive sensors, force sensors, etc. These sensors may be that which results in it having a high cost and it being complex to control. In terms of the self-sensing measurements, nowadays, many sensor types have been developed for numerous engineering applications. For instance, a piezoresistive force microsensor was designed and manufactured for measuring 3-axis forces [29]. In this study, eight piezoresistive strain gauges were attached on the surface of a silicon membrane. A Wheatstone bridge was employed to calculate the design of the sensor. The results achieved a high sensitivity of 1.03 mV/V/mN when it was under a normal force and another one of 1.6 mV/V/mN when it was under a tangential force. A type of piezoresistive pressure sensor was developed and fabricated in [30]. The cross-beam membrane, peninsula diaphragm, and a Wheatstone bridge were applied for this design. The achieved sensitivity of it was about 25.7 mV/kPa. Another force sensor was developed and simulated in [31]. The sensitivity that was measured was 0.8 mV/V/mN. In regard to measuring the displacement, a piezoresistive displacement sensor was designed for a microelectromechanical system (MEMS) nanopositioner [32]. In this design, a single-crystalline silicon was used as the structural material. In agricultural applications, a PET-based piezoresistive sensor was developed [33]. The achieved electrical sensitivity was 11 ppm/nm for sensing the proximal soil. Such piezoresistive pressure sensors can be found in popular applications in MEMS, e.g., for measurements of the force, fluid pressure, positions, and so on. The reasons for this are that the piezoresistive sensors have a good linearity and a high sensitivity. For the macro-sized engineering structures, strain gauge displacement sensors have been widely employed. A new type of strain gauge displacement sensor was developed for measuring the displacement or deformation of a beam/structure [34]. The strain gauge displacement sensor is ideal in practice due to its simple structure and low cost. It can be glued on the surfaces of the beams [35]. For detecting the displacement or deformation of the engineering structure, the strain gauge displacement sensor is also adopted.

Presently, researchers have concentrated on the four main parts for the 3-DOF flexure mechanisms, including its design, analysis, optimization, and control. For instance, a 3-DOF was designed and analyzed by a compliance matrix and a finite element method (FEM) [36]. They optimized the parameters of the stage to achieve a minimal input coupling ratio. Moreover, a 3-DOF manipulation was designed and analyzed in terms of considering the translational and angular displacements [37]. In addition, a 3-DOF mechanism was analyzed via a pseudo-rigid-body model [38]. However, the previously existing 3-DOF micropositioning stages have a complex structure, a large size, and three PZTs actuators. Furthermore, the further tracking of the travel/displacement of the stages is complicated because the stages need a displacement sensor and a force sensor. These lead to it being complex to control and it having a high cost. Motivated by the gaps in the literature review, the present study proposes a new design of an XYZ micropositioner, which is intended for probing the properties of biomedical samples in nanoindentation. The new novelty of the proposed XYZ micropositioner is that it can achieve the same stroke in the *X*, *Y*, and *Z* axes. Especially, the proposed micropositioner only uses two PZTs to make three motions in three axes. In addition, the proposed micropositioner can perform self-position sensing by embedding the strain gauge displacement sensor. The strain gauges-based sensor is adopted because it is easy for it to be attached to the strain gauge on the flexure hinges. Moreover, to improve the performances of the proposed micropositioner, an efficient optimizer with fewer parameters for the teaching–learning-based optimization was chosen to optimize the geometrical parameters [39].

The new contribution of this paper is to design and optimize a new XYZ micropositioner for biomedical sample probing application in the field of nanoindentation. The developed micropositioner can generate a large stroke in the X, Y, and Z directions. The displacements in these three axes are equally large. The safety factor and the displacement of the XYZ micropositioner are evaluated and optimized based on the Taguchi method, the response surface method, and teaching–learning-based optimization. The optimal solutions are evaluated through a finite element analysis. The output performances of the proposed micropositioner are compared to other designs.

## 2. Conceptual Design of XYZ Micropositioner with Embedded Displacement Sensor

### 2.1. Design of XYZ Micropositioner

The proposed micropositioner utilizes two PZT actuators to generate an input displacement. Specifically, the positioner is built with a monolithic structure that is linked by rigid joints and flexure hinges, which is composed of three main mechanisms: (i) a symmetrical four-lever displacement amplifier, (ii) a symmetrical parallel guiding mechanism, and (iii) a symmetrical parallel redirection mechanism. The parallel redirection mechanism consists of eight Z-shaped flexure leaf hinges to change the motion direction from the X and Y directions to the Z direction with almost equal approximate output values for the three directions. In addition, the parallel guiding mechanism consists of four flexure right-circular hinges that are combined with two rigid joints and two flexure leaf hinges in order to achieve a large workspace with small parasitic motion errors. Unlike the previous studies, the simultaneous input displacements in the X and Y directions generate three simultaneous output displacements in the X, Y, and Z directions. Especially, the Z directional movement is based on the Z-shaped flexure leaf hinges, which are considered in this research.

**Figure 1:**
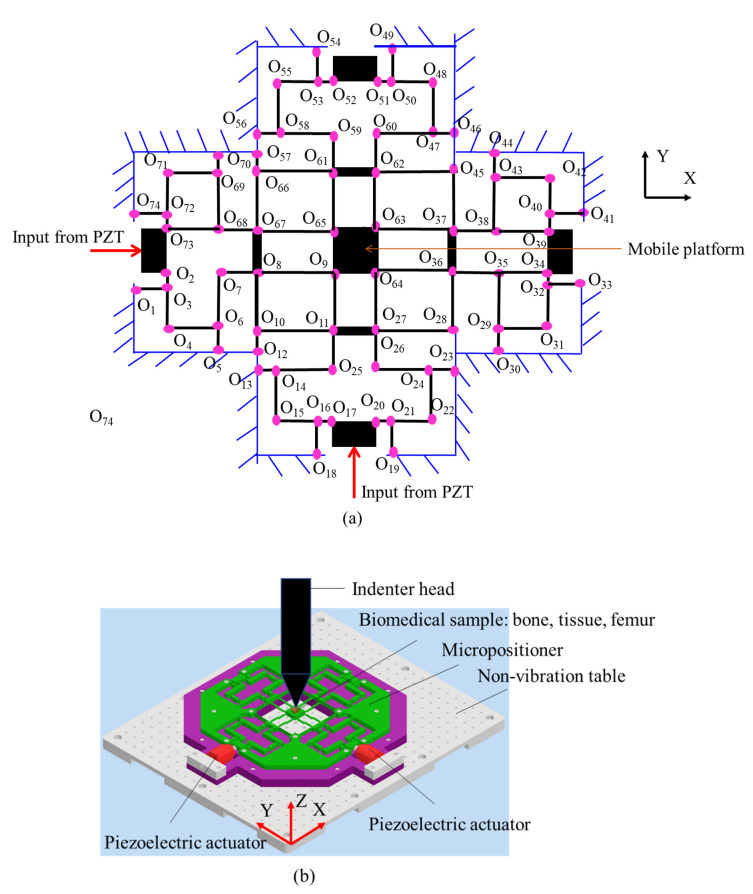
XYZ micropositioner for nanoindentation: (**a**) Kinematic scheme and (**b**) basic application for nanoindentation.

A kinematic scheme of the proposed XYZ micropositioner is illustrated in Figure 1a. The proposed micropositioner can be moved based on the kinematic joints O_1_ through to O_74_ which are coupled with links. It is operated based on two input PZTs actuators to generate the motion of the mobile platform. A basic application of the compliant XYZ micropositioner is used for positioning the biomedical specimens, e.g., artificial bones, tissues, and femurs, in a nanoindentation tester, as illustrated in Figure 1b. The biomedical sample is located on the proposed XYZ micropositioner, and the micropositioner is fixed on a non-vibration table. The mechanical properties of the sample, i.e., the Young’s modulus of the hardness, is probed by the head indenter. The accurate position of the sample is decided based on the movement of the micropositioner.

**Figure 2:**
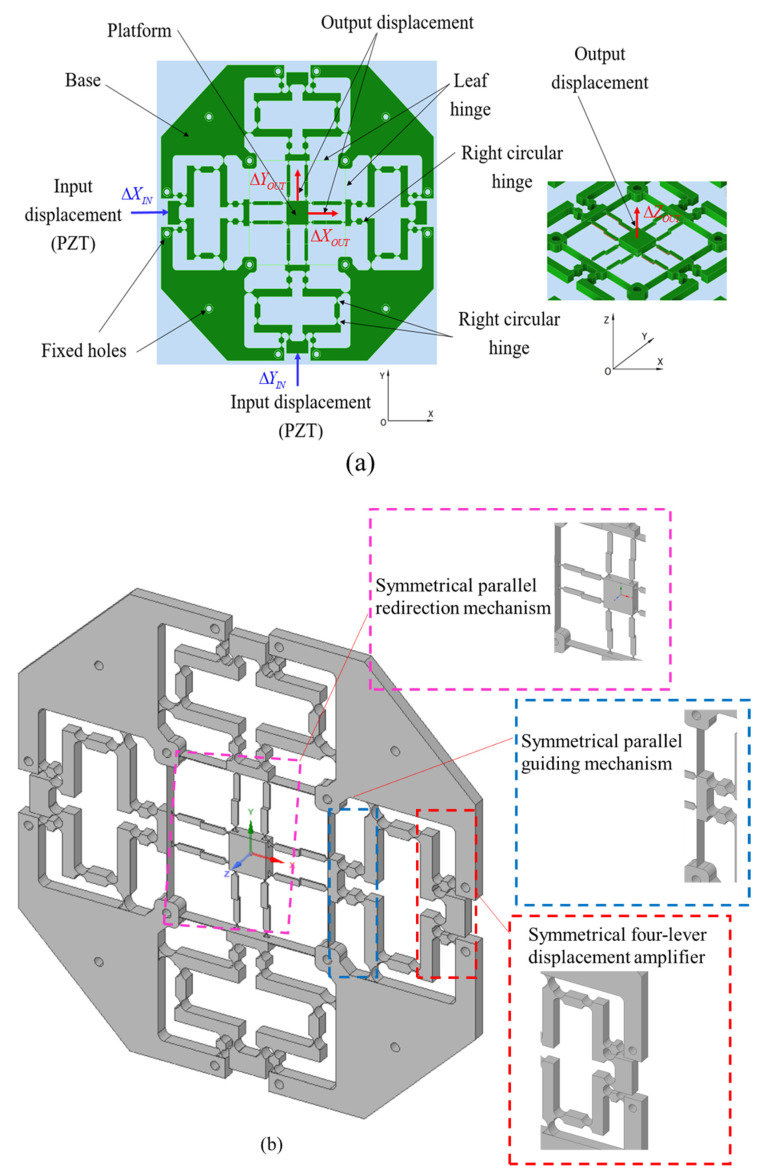
Design diagram of XYZ micropositioner: (**a**) design schematic and (**b**) detailed parts.

Figure 2a illustrates a design diagram of the proposed XYZ micropositioner. The detailed main parts such as the displacement amplifier, the parallel guiding mechanism, and the parallel redirection mechanism are given in Figure 2b.

**Figure 3:**
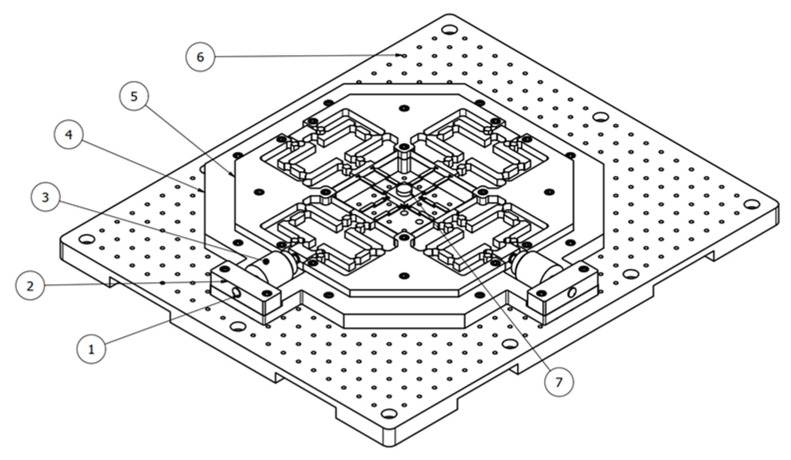
Assemble diagram of XYZ micropositioner: (1) preload screw, (2) PZT mounting plate, (3) PZT actuator, (4) intermediate plate, (5) prototype, (6) anti-vibration fixing plate, (7) specimen.

Figure 3 illustrates an assembly diagram of the XYZ micropositioner. It includes the following main components: (1) the preload screw, (2) the PZT mounting plate, (3) the PZT actuator, (4) the intermediate plate, (5) the prototype, (6) the anti-vibration fixing plate, and (7) the specimen. The prototype of the XYZ micropositioner is mounted on the intermediate plate. The two PZTs are fixed on the PZT mounting plate, and the preload screw is employed to adjust the PZT so that it is in contact with the input port of the platform. The proposed system is positioned on the anti-vibration table.

**Figure 4:**
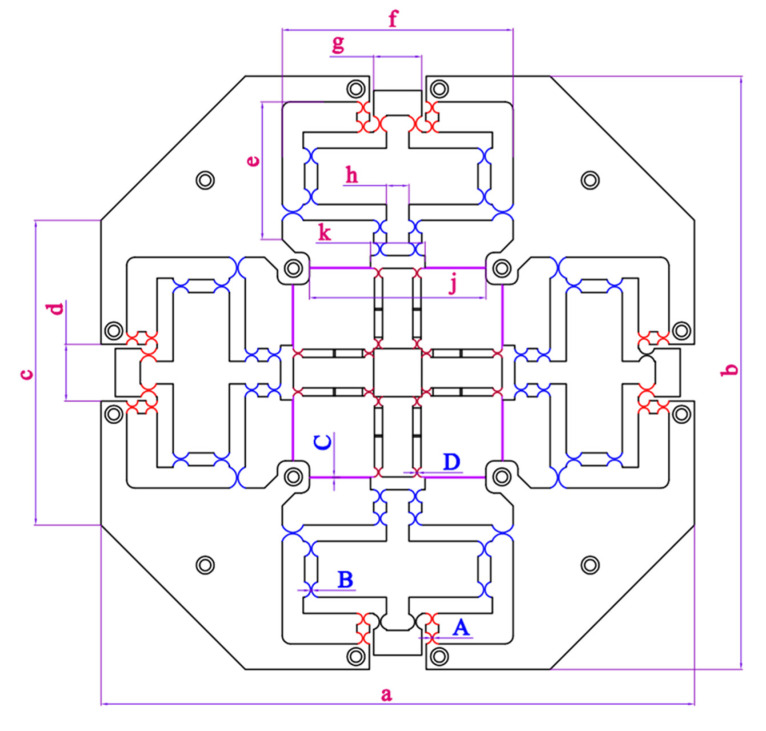
Basic parameters of XYZ micropositioner.

The main parameters of the XYZ micropositioner are given in Figure 4 and Table 1.

In this work, the prototype of the XYZ micropositioner is made using Al 7075-T651 whose properties comprise a density of 2810 Kg/m^3^, a Poisson ratio of 0.33, a yield stress of 503 MPa, and a Young’s modulus of 71,700 MPa. To achieve a monolithic structure, a light weight, a high precision, and less assembly, the wire electrical discharged machining (WEDM) technique is employed to create the proposed XYZ micropositioner.

In summary, a good micropositioner should offer a high resolution, a large amount of travel, a high safety factor, linearity, an efficient rate of power consumption, a compact size or a light weight, etc. In the present study, three key problems are pursued as follows: (i) This work only uses two PZTs actuators to actuate the X and Y directions, and the micropositioner can gain three output displacements in the X, Y, and Z directions with the same value. Unlike previous studies, many researchers have used three actuators, but this study only employs two actuators, which help to decrease the amount of power consumption. Additionally, the four-lever displacement amplifier is adopted to amend the magnitude of the travel, while a symmetrical parallel guiding mechanism was used to increase the stiffness as well as permit the linearity of the motions. The use of a parallel redirection mechanism is suggested to achieve the output displacement in the Z direction. The novelty of the proposed design is to develop a new planar micropositioner that can generate three simultaneous output displacements. This leads to a compact size and a lightweight device. In the proposed design, the leaf hinges ™ to produce a large amount of displacement, while the right-circular hinges were adopted to permit the positioning accuracy. (ii) A large amount of displacement and a high safety factor are chosen as the two main design targets. A large amount of displacement is expected to enlarge the locations of the specimen. It having a high safety factor is necessary for it to achieve a long working time and the safety of the design. In addition, to enhance these two design targets, this would be conducted using the later optimization process. (iii) A displacement sensor is embedded into the proposed micropositioner, which can decrease the displacement sensors during the measurement testing. It also reduces the complexity of its control in future work.

### 2.2. Determination Analysis of Embedded Displacement Sensor

**Figure 5:**
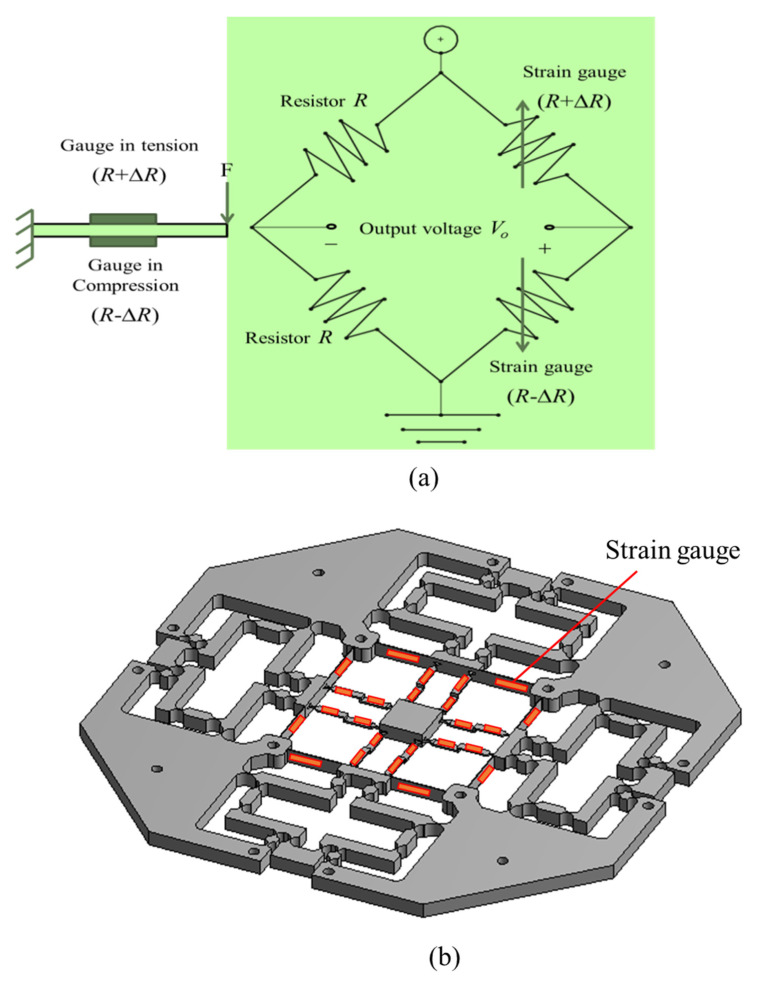
(**a**) Half-Wheatstone bridge circuit, and (**b**) strain gauge positions.

To measure the travel of the microplatform in three directions (X, Y, and Z), the strain gauges are glued onto the surfaces of the leaf hinges of the micropositioner where the max deformation appeared. The measurement process of the travel/displacement is based on a half-Wheatstone bridge circuit, as depicted in Figure 5a. The max deformation that occurs where the strain gauges are is shown in Figure 5b.

When the hinges of the micropositioner is deformed in a tension state, the resistance (*R*) is changed by an amount of Δ*R*. On the contrary, the *R* is decreased during compression. In the proposed sensor, the gauge factor is calculated using the following formula:

(1) $$G=\frac{\Delta R}{R\times \varepsilon },$$

where *G* notes the gauge factor and Δ*R* represents the resistance change, *R* depicts a nominal value of strain gauge, and *ε* notes the strain.

Then, the stress (*σ*) is defined by the following formula:

(2) σ=E×ε,

where *E* is Young’s modulus of the material.

The output voltage of the circuit is defined as follows:

(3) $$V_{o}=\frac{G\varepsilon }{2}\times V_{ex}=\frac{\Delta R}{2R}\times V_{ex},$$

where *V_o_* and *V_ex_* represent the circuit output and the excitation voltage, respectively.

From Equations (1)–(3), *V_o_* is recalculated by:

(4) $$V_{o}=\frac{G\sigma }{2E}\times V_{ex},$$

The exerted force (*F*) is related to the displacement (*δ*) of the leaf hinge as follows:

(5) F=K×δ,

where *F* is a force which exerted on the leaf hinge/beam, while *δ* notes the displacement of the leaf hinge.

Meanwhile, the force is related to the stress of the hinge by:

(6) $$\sigma =\frac{6Fl}{wt^{2}},$$

It is noted that the stiffness (*K*) of the hinge was calculated by:

(7) $$K=\frac{Ewt^{3}}{l^{3}},$$

where *t*, *l*, and *w* are the thickness, length, and width of the leaf hinge, respectively.

Lastly, the output voltage is redefined as follows:

(8) $$V_{o}=\frac{3V_{ex}Gt}{l^{2}}\times \delta =S\times \delta ,$$

The sensitivity of the strain gauge (*S*) is defined as:

(9) $$S=\frac{3V_{ex}Gt}{l^{2}},$$

During its real usage, the proposed micropositioner should offer a large amount of travel and a high resolution. A good design of the embedded displacement sensor of a device should ensure a large signal-to-noise (S/N) value. It means that a higher S/N value will reduce the sensing errors, while the resolution is enhanced. In order to improve the S/N value, the strain gauges should be attached around the positions of the flexure hinges where the maximum stress appears and is highly concentrated. Hence, the exact locations of strain gauges on the flexure hinges are critically important.

### 2.3. Initial Calculations

In the literature review, the previous studies have developed many 3-DOF micropositioning stages in which the X displacement differs from the Y displacement and the Z-displacement. In addition, the previous studies employed three actuators to generate the 3-DOF motion. Unlike the previous study, the present study proposed a new micropositioner that can generate three of the same output displacements, but it only used two actuators from the X and Y directions. A large amount of displacement/travel is desired in the proposed micropositioner. The large amount of travel is necessary for it to provide a wide range of practical applications.

Regarding the safety factor, the proposed micropositioner is designed by using the compliant mechanism. The motions of this mechanism are reflected in the elastic deformation of the flexure hinges/compliant joints whose thicknesses are very thin. The motion of the compliant mechanism is dependent on the material’s abilities, and the compliant mechanisms are only operated efficiently within the elastic limit of a used material. It means that the resulting stress inside the mechanism must be lower than the yield strength is of the used material. Otherwise, a failure will occur, e.g., plastic deformation, and especially, the fatigue life of the mechanism will be decreased. Therefore, to ensure the safe operation of it, the safety factor of the proposed micropositioner should be considered to be higher than 1.8 during the design synthesis phase.

In this study, the safety factor is desired to have a value of at least 1.8, and the output displacements in the X, Y, and Z directions are largest that they can be. An input displacement range from 77 µm to 80 µm is investigated. Using the Taguchi method for the four input parameters (*A*, *B*, *C*, and *D*), the L_9_ experimental number was built. Parameters *A*, *B*, *C*, and *D* can be named as design variables *X_A_*, *X_B_*, *X_C_*, and *X_D_*, respectively.

**Figure 6:**
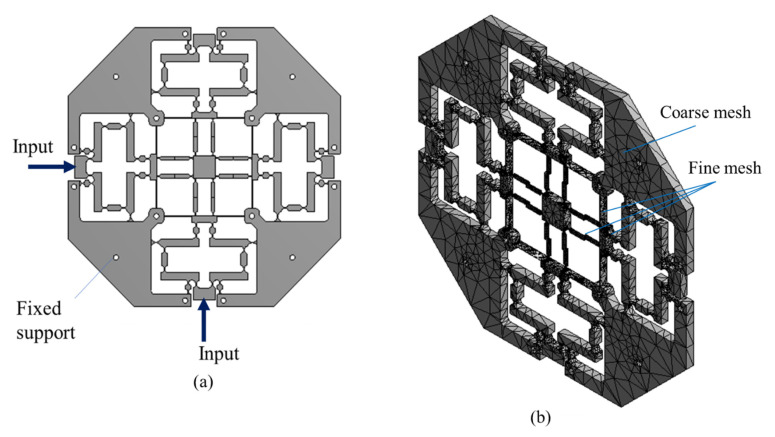
Boundary condition of XYZ micropositioner simulation: (**a**) Boundary condition, (**b**) mesh.

In order to limit the input displacement value for ensuring a safety factor value of 1.8, the prototype of the XYZ micropositioner is created using Inventor software, and the finite element analysis (FEA) simulation was performed using ANSYS 2019. The boundary condition is shown in Figure 6a. The fixed supports (A point) are located at the holes, while the two input displacements (X and Y direction) from the PZT are located along the X and Y directions, respectively. The output displacements are measured as depicted in Figure 2. The results are illustrated in Table 2, Table 3, Table 4 and Table 5. The model of the micropositioner is meshed, as it is given in Figure 6b. For the rigid links, i.e., where the rigid links are assumed to be ideally rigid, coarse meshes are employed. Meanwhile, the right-circular hinges and leaf hinges are meshed with the fine meshes because these hinges are deformable. The 10-node tetrahedral elements are employed in the mesh model.

**Figure 7:**
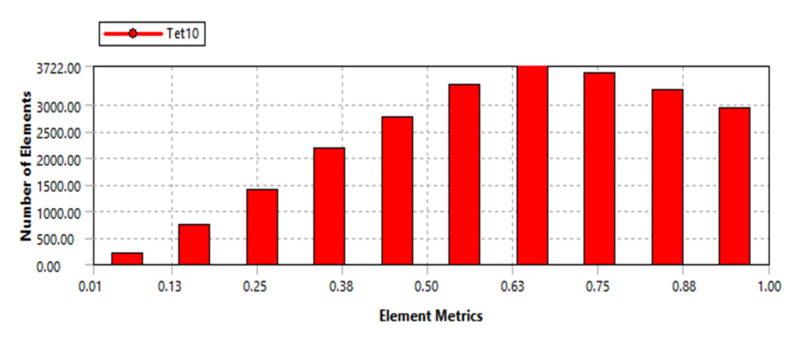
Meshing model of micropositioner: Meshed quality criteria using skewness.

The results of the meshing showed that the nodes have a value of 48,613 and the elements have a value of 24,038. To evaluate the mesh quality, skewness is adopted, and its average value is 0.62, as depicted in Figure 7. This value ensures that we have acquired a well-meshed model before performing the simulation.

Through the achieved results in Table 2, Table 3, Table 4 and Table 5, it can conclude that the displacements in the X, Y, and Z directions (as shown in Figure 2) are almost the same.

From the achieved results in Table 2, Table 3, Table 4 and Table 5, it determined that the appropriate input displacement of the two PZTs is 80 μm and this allows them to gain a large amount of travel. Additionally, the results of Table 2, Table 3, Table 4 and Table 5 showed that the stress values are still smaller than the yield strength of the Al material (503 MPa). This can ensure a safe operation for the proposed micropositioner in the elastic area.

**Figure 8:**
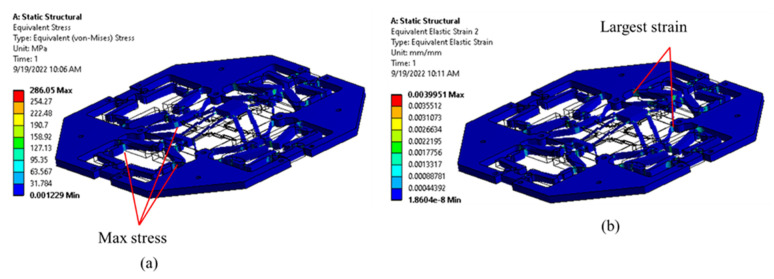
Simulation of XYZ micropositioner: (**a**) stress, and (**b**) strain.

In order to observe and determine the locations with a high concentration of the stress, the deformation, and the strain of the proposed micropositioner, in this part, we conducted a few FEA simulations using the ANSYS 2019R2 software. The results showed that the highest stress zones are found at the corners of the hinges, as shown in Figure 8a. To decrease the stress, the corners of the hinges were rounded. Meanwhile, the largest strain zones are distributed along the leaf hinges where the strain gauges would be glued on the positions of largest stress/strain in improving the sensing sensitivity, as seen in Figure 8b. The high stress appeared in the zone where the large strain occurred. In terms of the elastic limitation of the material, the stress can be calculated using Equation (2).

**Figure 9:**
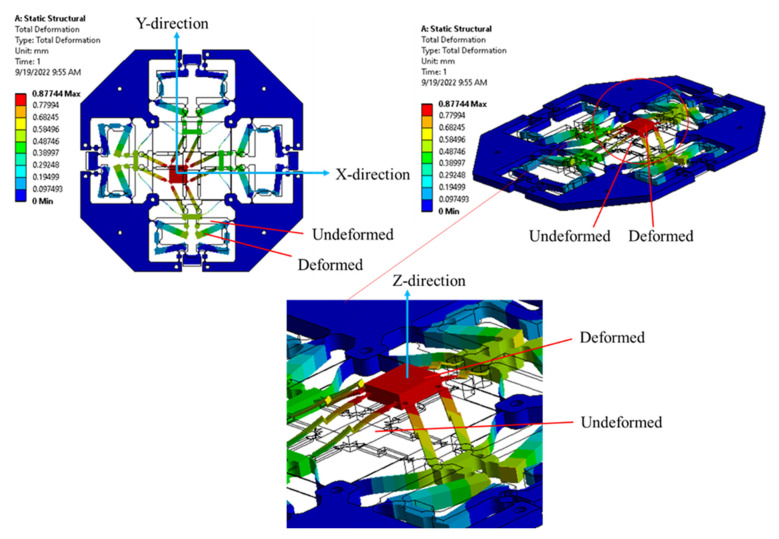
Deformation simulation of XYZ micropositioner.

Based on the deformation/deflection of hinges, the micropositioner is moved along the X, Y, and Z directions. Among the different flexure hinges, the leaf hinge is capable of providing a large amount of deformation. Meanwhile, the right-circular hinge with a smaller deformation is employed to perform a precision motion. Lastly, the deformation distribution of the proposed micropositioner is observed along the hinges, as depicted in Figure 9. The deformed and undeformed topology of the proposed micropositioner are also provided in this figure.

**Figure 10:**
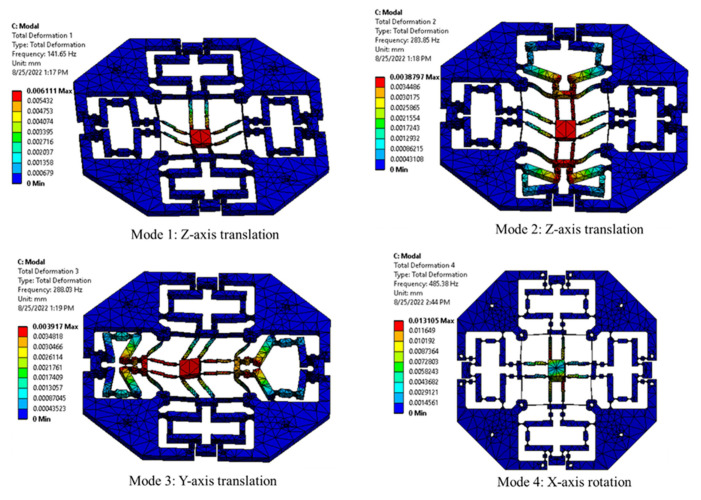
Dynamic performance of XYZ micropositioner with four of the first mode shapes.

In order to test the dynamic performance of the XYZ micropositioner, the dynamic simulation is carried out, as given in Figure 10. It has been found that the four first mode shapes are determined as follows: (i) the first mode is moved in the *Z*-axis (141.65 Hz), (ii) the second mode is displaced in the *Z*-axis as well (283. 85Hz), (iii) the third mode is translated in the *Y*-axis (288.03 Hz), and (iv) the fourth mode is rotated in the *X*-axis (485.38 Hz). The purpose of dynamic analysis is to determine the resonant frequencies. In practice, two PZTs are employed to generate two inputs for the micropositioner. One PZT was applied along the X direction, and the second PZT is applied along the Y direction, as shown in Figure 6. The reason for considering the dynamic response of the proposed device is that during the operation of it, the vibrations from PZTs actuators and other extra vibration sources may cause a resonant phenomenon when the frequency of the device is equal to the frequency of the actuators, especially the first natural frequency. Hence, the study on the dynamic response plays a vital role to avoid the unexpected resonances. Another reason for it is that the PZTs actuators often have a hysteresis property which will affect the device. So, the dynamic response is considered to discover how to enhance the response speed of the system.

With the benefit of the FEA simulation, this section provides an understanding of the dependence of various geometrical parameters on the overall sensor performance. In addition, the sensitivity of it in terms of the device dimensions and linearity is also determined.

**Figure 11:**
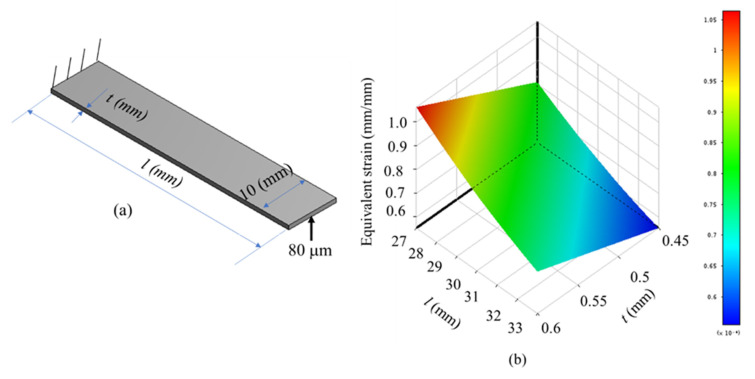
Influence of thickness and length of leaf hinge to the strain: (**a**) dimension of leaf hinge and (**b**) 3D plot.

From Equation (9), it can be observed that the sensitivity of the strain gauge-integrated sensor is dependent on the thickness (*t*) and the length (*l*) of leaf hinge. Meanwhile, the gauge factor is reflected in the strain in Equation (1). In this study, the strain gauges are glued onto the surfaces of the leaf hinges (see Figure 5). Hence, the sensitivity of the suggested sensor is evaluated by the FEA simulations of the leaf hinge (see Figure 11a). As shown in Figure 11b, an input displacement of 80 μm is applied to the free beam and another end is fixed. The results indicated that the strain value increased when the *t* parameter increased. On the contrary, the strain value decreased as when the *l* parameter was raised. The plot shows a linear relationship between the geometrical parameters of the leaf hinge and its strain value. Therefore, the proposed sensor behaves nearly linearity. The simulated sensitivity is about 0.21 V/mm.

## 3. Parameter Optimization of XYZ Micropositioner

### 3.1. Statement of Optimization Problem

The purposes of the proposed XYZ micropositioner are to maximize the safety factor (*F*_1_) and maximize the size of the Z direction output displacement (*F*_2_). The Z directional displacement is chosen due to the fact that the displacements in the three directions are almost same. The displacement, or the so-called travel, is considered as the design target, and it was as large as it could possibly be. When a large amount of travel of the micropositioner is achieved, it can move the specimens to various locations in the nanoindentation system. In addition, a high safety factor means that it having a long working time and its safety are ensured. In this study, the Taguchi method (TM) is combined with the response surface method (RSM) and TLBO to enhance the performances of the XYZ micropositioner. The optimizing process is performed using MATLAB 2017. The trouble of performing the optimization for the proposed XYZ micropositioner can be comprised as follows:

Find $X=\left[A,B,C,D\right]=\left[X_{A},{\;X}_{B},{\;X}_{C},{\;X}_{D}\right]$

Maximize the safety factor:

(10) $$F_{1}\left(X_{A},{\;X}_{B},{\;X}_{C},{\;X}_{D}\right),$$

Maximize the displacement:

(11) $$F_{2}\left(X_{A},{\;X}_{B},{\;X}_{C},{\;X}_{D}\right),$$

S.t.:

(12) $$\left\{\begin{array}{l} F_{1}\left(X_{A},{\;X}_{B},{\;X}_{C},{\;X}_{D}\right)\geq 1.8\\ F_{2}\left(X_{A},{\;X}_{B},{\;X}_{C},{\;X}_{D}\right)\geq 510\;\mu m \end{array}\right.,$$

Space of the design variables:

(13) $$\left\{\begin{array}{l} 0.8\;\mathrm{mm}\leq X_{A}\leq 0.9\;\mathrm{mm},\;0.7\;\mathrm{mm}\leq X_{B}\leq 0.8\;\mathrm{mm}\\ 0.55\;\mathrm{mm}\leq X_{C}\leq 0.65\;\mathrm{mm},\;0.6\;\mathrm{mm}\leq X_{D}\leq 0.7\;\mathrm{mm} \end{array}\right.,\;\;$$

where *F*_1_ and *F*_2_ are the objective responses. *X_A_*, *X_B_*, *X_C_*, and *X_D_* represent the design parameters *A*, *B*, *C*, and *D* of the micropositioner, respectively. Speccifically, *X_A_*, *X_B_*, *X_C_*, and *X_D_* note the thickness of the flexure right-circular hinge (lever 1), the thickness of the flexure right-circular hinge (lever 2), the thickness of the flexure leaf hinge (a guiding mechanism), and the thickness of Z-shaped flexure right-circular hinge (a guiding mechanism), respectively.

### 3.2. Proposed Methodology

**Figure 12:**
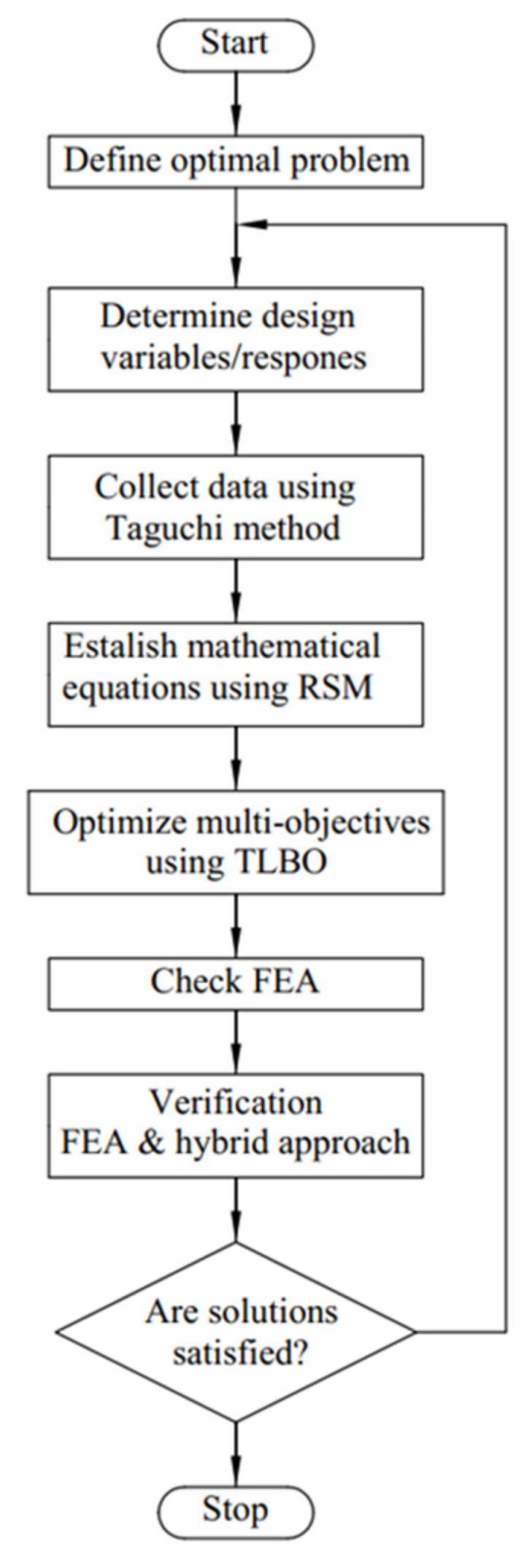
Flowchart of the combined optimization method.

In this research, an integration method diagram is suggested to improve the safety factor and the displacement of the XYZ micropositioner, as shown in Figure 12.

Firstly, the Taguchi method is exploited for planning numerical experiments. Secondly, the response surface method is utilized to build the regression form as follows.

(14) $$F_{j}=\beta_{0}+\sum_{i=1}^{n}\beta_{i}x_{i}+\sum_{i=1}^{n}\beta_{ij}x^{2}{}_{i}+\sum_{i=1}^{n-1}\sum_{j=i+1}^{n}\beta_{ij}x_{i}x_{j}+\varepsilon_{i}$$

where:

- *β_i_* (*i* = 0, 1, 2, …, *n*): the unknown coefficients;
- *β_ij_* (*i* < *j*): the collaboration coefficients;
- *x*_1_, *x*_2_,…, *x*_*n*_: the predicted variables;
- *ɛ*: an error.

Then, an analysis of variance (ANOVA) investigates the significance of inputs to outputs (the safety factor and the displacement).

According to the flowchart in Figure 12, the optimized solutions would be verified by performing a finite element analysis (FEA) using the ANSYS software.

### 3.3. Teaching–Learning-Based Optimization

The TLBO algorithm is a metaheuristic optimizer that is based on the teaching and learning technique. The teachers play a significant role in creating a good student. The leaners are considered as the population, and a design vector is a course. The teaching and learning are main strategies of TLBO [40].

#### 3.3.1. Teaching Operation

The teacher process has the main ideals:

- Looking for the teacher to achieve the best solution.
- Calculating the mean results of the learners (*M_j_,*_i_) in a specific course.
- The quality of the teacher has a strong effect on the students through the following formula.

(15) $$Dm_{j,k,i}=r_{j,i}\left(X_{j,kbest,i}-T_{F}M_{j,i}\right),\;$$

where *Dm*_*j*,*k*,*i*_ notes an raised mean value. *X*_*j*,*kbest*,*i*_ notes the best learner. *T_F_* is the teaching factor. *r*_*j*,*i*_ has a random value in [0, 1]. *T_F_* is either one or two. *T_F_* can be computed randomly by:

(16) $$T_{F}=round\left[1+rand\left(0,1\right)\left\{\left.2-1\right\}\right.\right].$$

The existing solution is then updated by:

(17) $$X_{j,k,i}^{\prime }=X_{j,k,i}+Dm_{j,k,i},$$

where $X_{j,k,i}^{\prime }$ notes an updated value of $X_{j,k,i}$. If the result is satisfied, it will be the input for the learner operation.

#### 3.3.2. Learning Operation

The learners can be performed among the students. With any iteration *i*, *U* learner is compared with a *V* learner ($X_{U,i}^{\prime }\neq X_{V,i}^{\prime }$) as follows:

(18) $$\left\{\begin{array}{l} X_{j,U,i}^{\prime \prime }=X_{j,U,i}^{\prime }+r_{j,i}\left(X_{j,U,i}^{\prime }-X_{j,V,i}^{\prime }\right),\;\mathrm{if}\;f\left(X_{U,i}^{\prime }\right)<f\left(X_{V,i}^{\prime }\right)\\ X_{j,U,i}^{\prime \prime }=X_{j,U,i}^{\prime }+r_{j,i}\left(X_{j,V,i}^{\prime }-X_{j,U,i}^{\prime }\right),\;\mathrm{if}\;f\left(X_{V,i}^{\prime }\right)<f\left(X_{U,i}^{\prime }\right) \end{array}\right..$$

**Figure 13:**
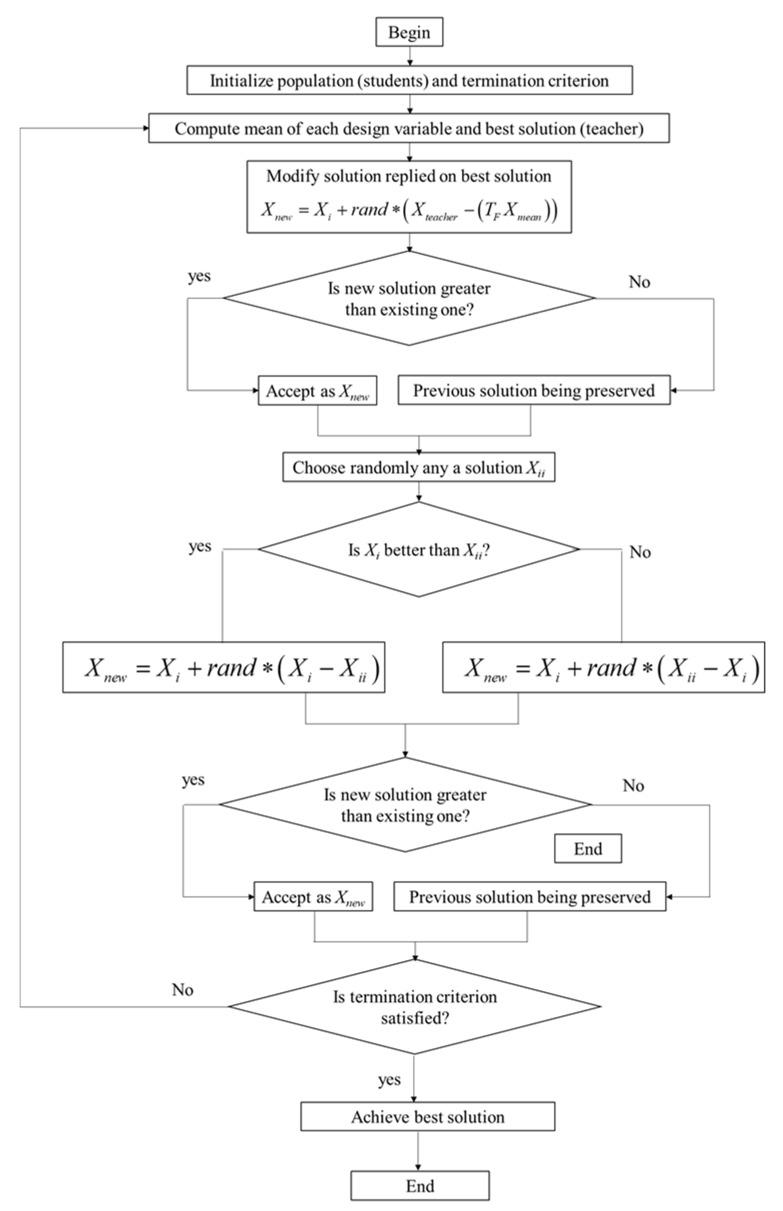
Flowchart of TLBO.

$X_{j,U,i}^{\prime \prime }$ is accepted as the objective value is better. A flowchart of the TLBO is demonstrated in Figure 13.

### 3.4. Influent Analysis of Design Parameters

The influences of the four design parameters (*A*, *B*, *C*, and *D*) are investigated in this section. From that, the designers can adjust one or a few parameters to improve the performances of the proposed micropositioner.

By using an ANOVA, the effects of the design factors to the safety factor and the displacement are analyzed, as illustrated in Table 6 and Table 7, respectively. As described in Table 6 in terms of *F*_1_, *B* was highest at 50.82%, while *D* was second highest at 36.18%. Meanwhile, *A* and *C* are relatively small, at 6.65% and 3.56%, respectively. Therefore, *B* and *D* should be substantially observed. As illustrated in Table 7 in terms of *F*_2_, *B* was highest at 92.61%, while *C* was relatively small at 6.27%. However, *D* and *A* are very small at 0.74% and 0.33%, respectively. Therefore, *B* should be significantly supervised. In Table 6 and Table 7, the P values of the parameters have a statistical significance.

### 3.5. Sensitivity Analysis

**Figure 14:**
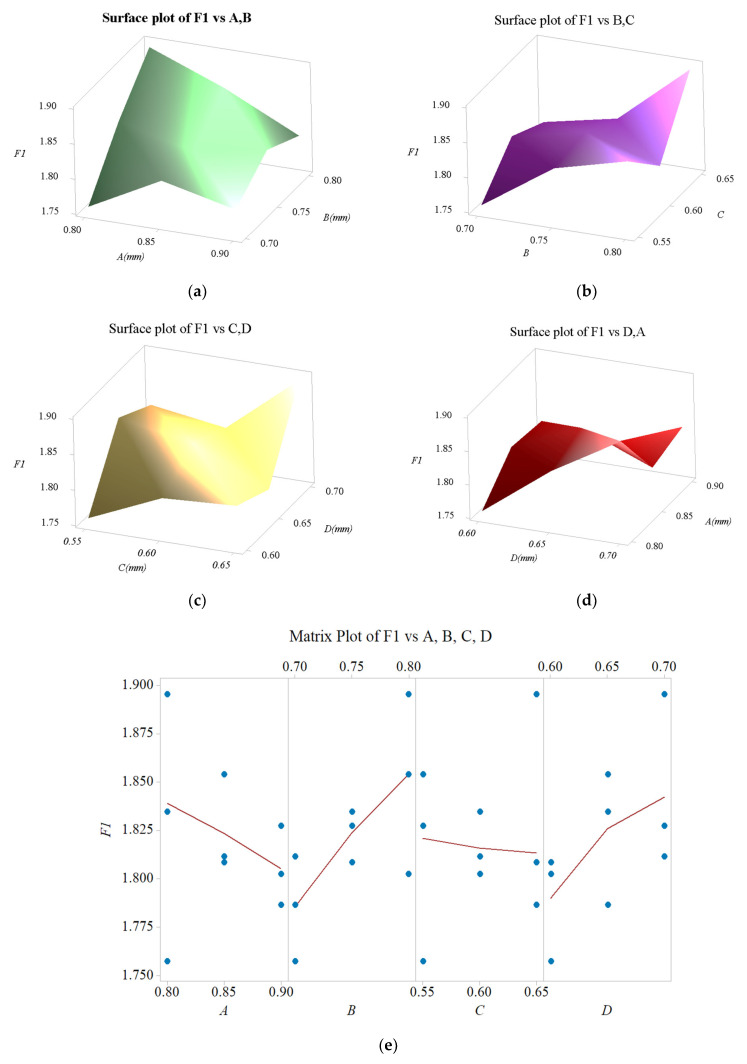
Influence diagram on safety factor: (**a**) of A and B, (**b**) of B and C, (**c**) of C and D, (**d**) of D and A, and (**e**) of A, B, C and D.

**Figure 15:**
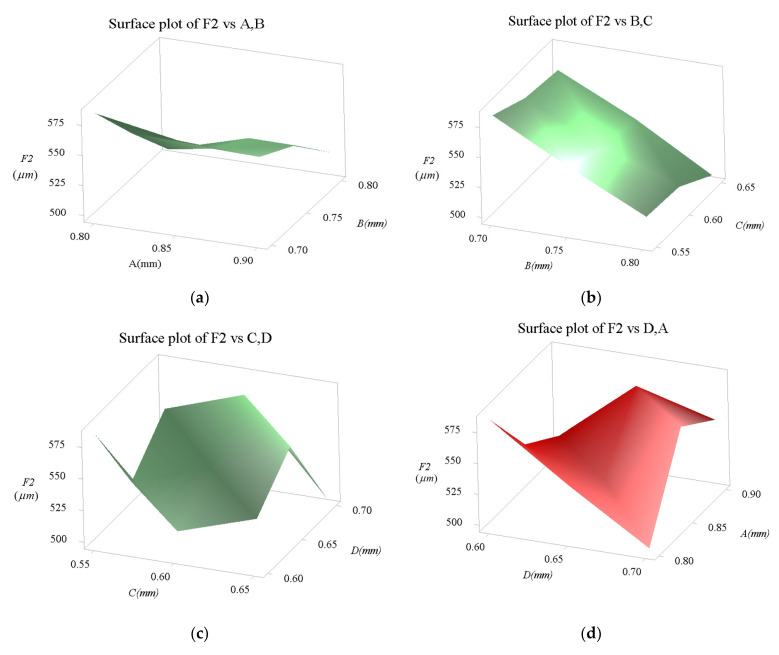

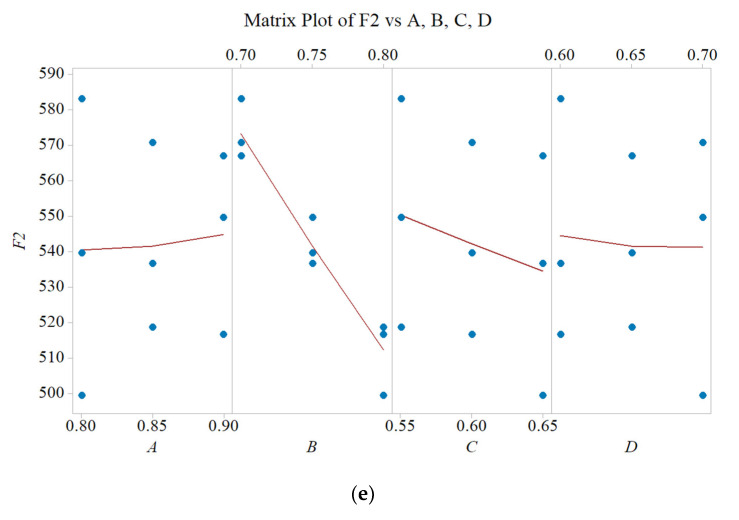
Influence diagram on output displacement: (**a**) of A and B, (**b**) of B and C, (**c**) of C and D, (**d**) of D and A, (**e**) of A, B, C and D.

The dependences of all of the performances of the proposed micropositioner with respect to all of the various geometrical parameters are discussed in this section. The results of the sensitivity test found that all of the parameters have a specific influence on the safety factor and the displacement, as given in Figure 14 and Figure 15, respectively. It means that any change in the parameter can lead a change in the response.

As illustrated in Figure 14, it is observed that the *F*_1_ value (safety factor) is quickly reduced when the parameter *A* is increased. On the contrary, when the parameter *B* is raised, the *F*_1_ value is also gradually increased. However, the *F*_1_ value experiences a slight change as the parameter *C* is varied. Lastly, it can be noted that the *F*_1_ value is very quickly increased. In conclusion, the four mentioned design parameters significantly influence the proposed micropositioner’s safety factor.

The results in Figure 15 show that the *F*_1_ value experiences little change when the *A* factor is raised. Especially when the *B* factor is increased, the *F*_1_ value is very quickly decreased. Meanwhile, the *F*_1_ value experiences little change as two factors *C* and *D* are varied. Based on the results of the sensitive study in Figure 11 and Figure 12, it indicated that all of the parameters have specific effects on the performances of proposed mechanism. The investigation of parametric sensitivity is an important step in determining the main design parameters in the optimization implementation.

### 3.6. Results of Optimization

Based on the data from Table 4, the regression equations for the safety factor and the output displacement, respectively, were obtained as follows:

(19) $$\begin{array}{ll} F_{1}= & -2.847+4.716*A+3.945*B-2.403*C+4.730*D\\ & -2.913*A^{2}-2.193*B^{2}+2.147*C^{2}-3.213*D^{2} \end{array}$$

(20) $$\begin{array}{ll} F_{2}= & 1583-334.9*A-1025*B-183.8*C-545.0*D\\ & +218.7*A^{2}+269.7*B^{2}+18.67*C^{2}+376.7*D^{2} \end{array}$$

The optimized values are at *A* = 0.9 mm, *B* = 0.8 mm, *C* = 0.57 mm, and *D* = 0.7 mm, and *F*_1_ = 1.85 and *F*_2_ = 515.7278 µm. The results were satisfied and applicable for the nanoindentation device and the micropositioning system.

## 4. Verification and Comparison

The optimal values (*A* = 0.9 mm, *B* = 0.8 mm, *C* = 0.57 mm, and *D* = 0.7 mm) were employed for designing the 3D model. The anticipated results were compared with the FEA ones. It was noted that the verification errors are very small, as illustrated in Table 8. Consequently, the forecasted results are a reliable confirmation according to the verified results.

Compared with the existing XYZ micropositioning stages, the output displacement/stroke of the developed XYZ micropositioner was used in the comparison, as provided in Table 9.

From Table 9, it can be observed that the workspace of the proposed XYZ micropositioner is much larger than that of the previously existing micropositioning stages. In comparison to the [23,26], the strokes in the *X*, *Y*, and *Z*-axes are almost the same. This is an advantage of XYZ stage which can permit the simple control of it. In addition, the designs in [19,21,25,28] have three different strokes in three respective axes, which can result in a complexity in the practice of its control and usage. Moreover, the proposed design of the XYZ micropositioner only needs two PZTs actuators to generate three motions. On the contrary, the previous designs of stages have required three PZTs actuators. Hence, we can conclude that the existing designs have a relatively high cost, while the presented design of this study offers a low cost. Lastly, another advantage of the proposed micropositioner is that it can be fabricated in a planar by using WEDM.

## 5. Conclusions

This study presented the design, analysis, and optimization of a new XYZ micropositioner. In the design phase, the proposed micropositioner was built by a combination of a symmetrical four-lever displacement amplifier, a symmetrical parallel guiding mechanism, and a symmetrical parallel redirection mechanism. The parallel redirection mechanism with Z-shaped hinges was built to gain the Z directional displacement. In the parallel guiding mechanism, four flexure right-circular hinges were combined with two rigid joints and two flexure leaf hinges to achieve the large X and Y directional displacement values. The symmetrical four-lever displacement amplifier was created to amend the magnitude of the travel of the micropositioner. The proposed micropositioner was integrated with the strain gauge to measure the displacement or force during operation. To establish the modeling of the performances of the proposed micropositioner, the Taguchi-based response surface method was employed. Then, the geometrical factors of the XYZ micropositioner were optimized by the TLBO optimizer.

The results of this paper were covered as follows. Through FEA initial simulations, the results determined an appropriate input displacement of 80 μm for two input ports in the X and Y directions. The output displacements in three X, Y, and Z directions are almost equal. By using the TLBO algorithm, the optimal design parameters were determined, which were *A* = 0.9 mm, *B* = 0.8 mm, *C* = 0.57 mm, and *D* = 0.7 mm. The optimized safety factor and the displacement were determined to be about 1.8364 and 516.58 µm, respectively.

The limitations of this study can be summarized as follows: (i) Although the designed micropositioner has a merit of achieving the three output motions simultaneously, the size of micropositioner should be compact so that it can be integrated into the SEM device for a practical micro/nanoindentation procedure to take place. (ii) Because the lever amplification type was employed, the size of the micropositioner was still large. A few new amplifiers should be developed to alternative the existing amplifier. (iii) This design has three output motions which move simultaneously, and while this is a merit of this design, it also means that the control of it is relatively complex. (iv) Meanwhile, this design is a novel idea for achieving three motions (X, Y, and Z) from two directions (X and Y). Actually, this design also generates several difficulties such as controlling the positioning locations, simultaneously. However, this problem can be solved easily by compensation by another actuator if the fabrication process is not accurate. In addition, the compensation that is required to obtain the position accuracy is relatively small. Therefore, this novel design will reduce the time that is needed for accurate positioning and be a potential design for future research in the compliant positioning field.

In a future study, the prototypes of the XYZ micropositioner will be fabricated by using WEDM. The experimental verifications will be carried out to validate the predicted performances of the micropositioner. Additionally, the sensitivity and resolution of the suggested sensor will be measured. The proposed micropositioner will be attached in the micro/nanoindentation tester to probe the mechanical properties of biomedical samples.

## Figures and Tables

**Table 1:** Dimensions of the XYZ micropositioner.

Symbol	Value	Symbol	Value	Unit
*a*	370	*h*	14	mm
*b*	370	*j*	110	mm
*c*	190	*k*	34	mm
*d*	35	*A*	0.8 ≤ *A* ≤ 0.9	mm
*e*	86	*B*	0.7 ≤ *B* ≤ 0.8	mm
*f*	144	*C*	0.55 ≤ *C* ≤ 0.65	mm
*g*	30	*D*	0.6 ≤ *D* ≤ 0.7	mm

**Table 2:** Numerical results (Input displacement of 77 µm).

No.	Input Displacement (µm)	*X_A_*	*X_B_*	*X_C_*	*X_D_*	*F*_1_	*F*_2_ (µm)	Stress (MPa)
1	77	0.8	0.7	0.55	0.6	1.8029	X: 568.86 Y:567.8 Z: 568.33	278.99
2	77	0.8	0.75	0.6	0.65	1.8822	X: 525.54 Y: 526.24 Z: 525.89	267.24
3	77	0.8	0.8	0.65	0.7	1.9447	X: 486.12 Y: 487.44 Z: 486.78	258.65
4	77	0.85	0.7	0.6	0.7	1.8584	X: 556.19 Y: 556.4 Z: 556.295	270.66
5	77	0.85	0.75	0.65	0.6	1.8553	X: 522.57 Y: 523.41 Z: 522.99	271.12
6	77	0.85	0.8	0.55	0.65	1.902	X: 504.49 Y: 506.45 Z: 505.47	264.46
7	77	0.9	0.7	0.65	0.65	1.833	X: 552.78 Y: 552.39 Z: 552.585	274.41
8	77	0.9	0.75	0.55	0.7	1.8745	X: 535.28 Y: 536.05 Z: 535.665	268.34
9	77	0.9	0.8	0.6	0.6	1.8492	X: 503.05 Y: 503.93 Z: 503.49	272.01

Note: *F*_1_ and *F*_2_ represent the safety factor and the output displacement, respectively.

**Table 3:** Numerical results (Input displacement of 78 µm).

No.	Input Displacement (µm)	*X_A_*	*X_B_*	*X_C_*	*X_D_*	*F*_1_	*F*_2_ (µm)	Stress (MPa)
1	78	0.8	0.7	0.55	0.6	1.7798	X: 576.25 Y:575.18 Z: 575.715	282.62
2	78	0.8	0.75	0.6	0.65	1.8581	X: 532.47 Y: 533.07 Z: 532.77	270.71
3	78	0.8	0.8	0.65	0.7	1.9197	X: 492.43 Y: 493.77 Z: 493.1	262.02
4	78	0.85	0.7	0.6	0.7	1.8345	X: 563.42 Y: 563.63 Z: 563.525	274.19
5	78	0.85	0.75	0.65	0.6	1.8315	X: 529.36 Y: 530.21 Z: 529.785	274.64
6	78	0.85	0.8	0.55	0.65	1.8776	X: 511.04 Y: 513.03 Z: 512.035	267.9
7	78	0.9	0.7	0.65	0.65	1.8095	X: 559.96 Y: 559.56 Z: 559.76	277.98
8	78	0.9	0.75	0.55	0.7	1.8505	X: 542.23 Y: 543.01 Z: 543.12	271.82
9	78	0.9	0.8	0.6	0.6	1.8255	X: 509.58 Y: 510.48 Z: 510.035	275.54

Note: *F*_1_ and *F*_2_ represent the safety factor and the output displacement, respectively.

**Table 4:** Numerical results (Input displacement of 79 µm).

No.	Input Displacement (µm)	*X_A_*	*X_B_*	*X_C_*	*X_D_*	*F*_1_	*F*_2_ (µm)	Stress (MPa)
1	79	0.8	0.7	0.55	0.6	1.7573	X: 583.64 Y:582.55 Z: 583.095	286.23
2	79	0.8	0.75	0.6	0.65	1.8345	X: 539.29 Y: 539.9 Z: 539.595	274.19
3	79	0.8	0.8	0.65	0.7	1.8954	X: 498.74 Y: 500.1 Z: 499.42	265.38
4	79	0.85	0.7	0.6	0.7	1.8113	X: 570.64 Y: 570.85 Z: 570.745	277.7
5	79	0.85	0.75	0.65	0.6	1.8083	X: 536.14 Y: 537.01 Z: 536.575	278.16
6	79	0.85	0.8	0.55	0.65	1.8539	X: 517.59 Y: 519.61 Z: 518.68	271.32
7	79	0.9	0.7	0.65	0.65	1.7866	X: 567.13 Y: 566.74 Z: 566.935	281.54
8	79	0.9	0.75	0.55	0.7	1.8271	X: 549.18 Y: 549.97 Z: 549.575	275.3
9	79	0.9	0.8	0.6	0.6	1.8024	X: 516.12 Y: 517.02 Z: 516.66	279.07

Note: *F*_1_ and *F*_2_ represent the safety factor and the output displacement, respectively.

**Table 5:** Numerical results (Input displacement of 80 µm).

No.	Input Displacement (µm)	*X_A_*	*X_B_*	*X_C_*	*X_D_*	*F*_1_	*F*_2_ (µm)	Stress (MPa)
1	80	0.8	0.7	0.55	0.6	1.7353	X: 591.03 Y:589.92 Z: 590.475	289.86
2	80	0.8	0.75	0.6	0.65	1.8116	X: 546.12 Y: 546.74 Z: 546.43	277.66
3	80	0.8	0.8	0.65	0.7	1.8717	X: 505.06 Y: 506.43 Z: 508.43	268.74
4	80	0.85	0.7	0.6	0.7	1.7887	X: 577.86 Y: 578.08 Z: 577.97	281.21
5	80	0.85	0.75	0.65	0.6	1.7857	X: 542.93 Y: 543.8 Z: 543.365	281.68
6	80	0.85	0.8	0.55	0.65	1.8307	X: 524.14 Y: 526.19 Z: 525.165	274.76
7	80	0.9	0.7	0.65	0.65	1.7643	X: 574.31 Y: 573.91 Z: 574.11	285.1
8	80	0.9	0.75	0.55	0.7	1.8042	X: 556.13 Y: 556.93 Z: 556.53	278.79
9	80	0.9	0.8	0.6	0.6	1.7799	X: 522.65 Y: 523.57 Z: 523.11	282.6

Note: *F*_1_ and *F*_2_ represent the safety factor and the output displacement, respectively.

**Table 6:** ANOVA for safety factor.

Source	DF	Seq SS	Contribution	Adj SS	Adj MS	*p*-Value
Model	8	0.012663	100.00%	0.012663	0.001583	significant
Linear	4	0.012310	97.21%	0.012310	0.003078	significant
*A*	1	0.000843	6.65%	0.000843	0.000843	significant
*B*	1	0.006435	50.82%	0.006435	0.006435	significant
*C*	1	0.000451	3.56%	0.000451	0.000451	significant
*D*	1	0.004582	36.18%	0.004582	0.004582	significant
Square	4	0.000353	2.79%	0.000353	0.000088	significant
A*A	1	0.000106	0.84%	0.000106	0.000106	significant
B*B	1	0.000060	0.47%	0.000060	0.000060	significant
C*C	1	0.000058	0.45%	0.000058	0.000058	significant
D*D	1	0.000129	1.02%	0.000129	0.000129	significant
Error	0	-	-	-	-	
Total	8	0.012663	100.00%			

**Table 7:** ANOVA for displacement.

Source	DF	Seq SS	Contribution	Adj SS	Adj MS	*p*-Value
Model	8	6227.22	100.00%	6227.22	778.40	significant
Linear	4	6223.94	99.95%	6223.94	1555.98	significant
*A*	1	20.39	0.33%	20.39	20.39	significant
*B*	1	5766.93	92.61%	5766.93	5766.93	significant
*C*	1	390.75	6.27%	390.75	390.75	significant
*D*	1	45.87	0.74%	45.87	45.87	significant
Square	4	3.28	0.05%	3.28	0.82	significant
A*A	1	0.60	0.01%	0.60	0.60	significant
B*B	1	0.91	0.01%	0.91	0.91	significant
C*C	1	0.00	0.00%	0.00	0.00	significant
D*D	1	1.77	0.03%	1.77	1.77	significant
Error	0	-	-	-	-	
Total	8	6227.22	100.00%			

**Table 8:** Error between anticipated results and verifications.

Characteristics	Anticipation	Confirmation	Error (%)
*F*_1_	1.8548	1.8364	1.001
*F*_2_ (mm)	515.7278	516.58	0.165

**Table 9:** Comparison of the workspace of proposed XYZ micropositioner with the previously existing XYZ micropositioning stages.

XYZ Micropositioner	Workspace (X, Y, Z-Travels)	DOF	Number of Actuators
This design	516.58 μm × 516.58 μm × 516.58 μm	XYZ	2
[19]	19 μm × 120 μm × 1 μm	XYZ	3
[21]	165.8 μm × 5.4 μm × 6.5 μm	XYZ	3
[23]	112 μm × 112 μm × 112 μm	XYZ	3
[25]	10.39 μm × 15.43 μm × 15.55 μm	XYZ	3
[26]	141 μm × 141 μm × 141 μm	XYZ	3
[28]	128.1 μm × 131.3 μm × 17.9	XYZ	3

## Data Availability

The data used to support the findings of this study are included within the article.
